# Effect of Calcium Dobesilate in Preventing Contrast-Induced Nephropathy in Patients with Diabetes and Chronic Kidney Disease

**DOI:** 10.6061/clinics/2021/e2942

**Published:** 2021-10-05

**Authors:** Hao Zhang, Shao-Hua Guo, Zheng-Kai Xue, Ya-Ru Zhang, Jia-Rui Wang, Jing-Jin Che, Tong Liu, Hua-Yue Tao, Guang-Ping Li, Seung-Woon Rha, Swapnil-Zaman Ashraful-Haque, Kang-Yin Chen

**Affiliations:** I Tianjin Key Laboratory of Ionic-Molecular Function of Cardiovascular disease, Department of Cardiology, Tianjin Institute of Cardiology, The Second Hospital of Tianjin Medical University, Tianjin 300211, China.; II Information Department, the Second Hospital of Tianjin Medical University, Tianjin 300211, China.; III Cardiac Center, Korea University Guro Hospital, Seoul 152703, Korea.

**Keywords:** Calcium dobesilate, Contrast-Induced Acute Kidney Injury, Diabetes Mellitus, Chronic Renal Insufficiency, Coronary Angiography

## Abstract

**OBJECTIVES:**

This study assessed the protective effect of calcium dobesilate against contrast-induced nephropathy (CIN) after coronary angiography (CAG) or percutaneous coronary intervention (PCI) in patients with diabetes and chronic kidney disease (CKD).

**METHODS:**

A total of 130 patients with diabetes and CKD estimated glomerular filtration rate: 30-90 mL/min/1.73m^2^ were enrolled and included in the analysis. They were divided into experimental (n=65) and control groups (n=65). Patients in the experimental group were administered oral calcium dobesilate (500 mg) three times daily for 2 days before and 3 days after the procedure. The serum creatinine (SCr), cystatin C (Cys C), and neutrophil gelatinase-associated lipocalin (NGAL) levels were measured before and after the procedure.

**RESULTS:**

The mean SCr level at 24h after the procedure was found to be significantly lower in the experimental group than in the control group (79.1±19.6 μmol/L *vs*. 87.0±19.3 μmol/L, *p*=0.023). However, the Cys C and NGAL levels were not significantly different between the two groups at all measurement time points (all *p*>0.05). The incidence of CIN defined by the SCr level was significantly lower in the experimental group than in the control group (3 [4.6%] *vs*. 13 [20.0%], *p*=0.017). However, the incidence of CIN defined by the Cys C level was not statistically different between the two groups (7 [10.8%] *vs*. 7 [10.8%], *p*=1.000).

**CONCLUSIONS:**

This study revealed that calcium dobesilate has no preventive effect against CIN in patients with diabetes and CKD.

## INTRODUCTION

Iodinated contrast medium (CM) is widely used with the development of coronary artery intervention therapies such as coronary angiography (CAG) and percutaneous coronary intervention (PCI). Contrast-induced nephropathy (CIN) is a critical complication of CM. CIN is the third most common cause of hospital-acquired acute kidney failure. The incidence of CIN in the general population is approximately 3% (1). However, the risk factors for CIN are diabetes mellitus, chronic renal dysfunction, advanced age, and congestive heart failure (2,3). The incidence of CIN in patients with diabetes can reach up to 29.4% (4). CIN can increase the in-hospital costs and duration as well as lead to hemodialysis and death (5,6). In addition, the incidence of long-term cardiovascular adverse events in patients with CIN is far higher than that in patients without CIN (7). Thus, CIN has become a hot topic in both the kidney and heart research fields.

Intravenous hydration therapy can reduce the incidence of CIN; hence, hydration has become the most widely used method for preventing CIN (8). In addition to hydration, N-acetylcysteine, statins, and other drugs are used to prevent CIN (9-11), although the effects of these drugs are controversial and require confirmation. However, the effects of these agents on CIN remain unclear in patients with diabetes and chronic kidney disease (CKD). Calcium dobesilate, a vascular protective drug, is used for the treatment of microvascular diseases (12) such as diabetic retinopathy and diabetic nephropathy. Some studies have suggested that calcium dobesilate can reduce blood viscosity, platelet activity, and capillary permeability (13), as well as alleviate microcirculatory and hemorheological abnormalities (14). The pathogenesis of CIN might be related to vasoconstriction, inflammation, and cytotoxicity caused by contrast agents (15-18). Hence, we speculated that calcium dobesilate might play a preventive role in CIN through the aforementioned mechanism. Thus, this study assessed whether calcium dobesilate has a potential protective effect against CIN in patients receiving CAG or PCI.

## MATERIALS AND METHODS

This study was a prospective, randomized, single-blind, controlled trial. This study was funded by the Chinese Cardiovascular Association V.G Foundation (No. 2017-CCA-VG-021) and the clinical medical research project of the Second Hospital of Tianjin Medical University (2020LC12). All patients signed an informed consent form before participating in the study. This study was conducted in accordance with the Declaration of Helsinki, a statement of ethical principles to provide guidance to physicians and other participants in medical research involving human subjects (19).

### Study population

We included 160 patients with diabetes who had been diagnosed with stage 2-3 CKD (estimated glomerular filtration rate [eGFR] was between 30 and 90 mL/min/1.73m^2^) and scheduled for coronary angiography between April 2017 and December 2019. The exclusion criteria were as follows: (1) acute myocardial infarction requiring primary PCI; (2) severe heart failure, above New York Heart Association (NYHA) class III; (3) infectious diseases, severe hepatic insufficiency, and acute cerebrovascular diseases; (4) severe coagulation disorder and active bleeding; (5) diabetic ketoacidosis, lactic acidosis, or other acute complications of diabetes; (6) other clinical conditions causing renal function damage; (7) pregnancy or malignant tumor; and (8) allergy to calcium dobesilate.

### Sample size calculation

Based on the study by Gruberg et al. (20), the incidence of CIN in patients with diabetes and CKD following CAG was reported to be 37%. This study was the first to evaluate the preventive value of calcium dobesilate; hence, we assumed that the preventive effect of calcium dobesilate was not less than that of N-acetylcysteine. We used the incidence of N-acetylcysteine, which was 11% according to the study by Albarazy et al. (21), to calculate the sample size with a power of 80% and *α*=0.05, with a 95% confidence interval (CI). Consequently, 40 patients in each group will be required to show a significant result (22). Considering the loss and refusal to follow-up, 80 patients were required in each group.

### Study protocol

The patients were allocated 1:1 into experimental and control groups according to a systematic randomization computer-generated list by an independent medical staff who was not involved in the study. All patients received the standard treatment of aspirin and clopidogrel for coronary heart disease. In addition, other treatments such as statins, angiotensin-converting enzyme inhibitors, angiotensin II receptor blockers, β blockers, nitrates, calcium channel blockers, and diuretics were used according to the underlying diseases of the patients. The experimental group received an oral dose of 500 mg calcium dobesilate (Hainan Linheng Pharmaceutical Co., Ltd., Haikou, China) three times daily for 2 days before and 3 days after the procedure. All patients were administered the standard hydration therapy: intravenous infusion of 0.5-1 mL/kg/h normal saline 6-12h before the procedure and up to 12-24h after the procedure. Interventional cardiologists performed all CAG or PCI procedures using the standard method according to the latest guidelines. All patients received a low osmolar CM (Iohexol, GE Healthcare, Shanghai, China).

### Data and sample collection

Demographic data such as age, sex, height, weight, medication history were collected. The left ventricular ejection fraction was measured by echocardiography using the modified biplane Simpson’s method. Baseline blood and urine tests were routinely performed in all patients during admission. Blood samples were collected from all patients at 24 and 72h after the procedure to measure the levels of blood urea nitrogen (BUN), serum creatinine (SCr), serum uric acid (UA), and serum cystatin C (Cys C). The SCr level was measured using the sarcosine oxidase method (BS2000M, Mindray Medical International Co., Ltd., Shenzhen, China). Urine samples were collected at 6 and 48h after CAG or PCI for the measurement of neutrophil gelatinase-associated lipocalin (NGAL) using the enzyme-linked immunosorbent assay method (XLPCC, Shanghai Xinle Bio-technology Co., Ltd, Shanghai, China). Urine samples were stored at −80°C until analysis. The eGFR in each patient was calculated using the Modification of Diet in Renal Disease Formula (23). Hypertension was defined as a blood pressure of ≥140/90 mmHg or current use of antihypertensive medication. Mehran CIN-risk scores were initially developed for the simple evaluation of an individual patient’s risk of developing CIN. It included eight clinical and procedural variables: hypotension, intra-aortic balloon pump, congestive heart failure, CKD, diabetes, age >75 years, anemia, and volume of CM (24).

### Endpoint

The primary endpoint of the study was the incidence of CIN, which was defined as an increase of >44 μmol/L or >25% in the level of SCr (25) or of 25% in the level of basal Cys C after 48-72 h of CAG or PCI (26).

### Statistical analyses

Statistical analyses were performed using Statistical Package for Social Sciences 22.0 version (SPSS, SPSS. Inc., Chicago, IL, USA). Continuous variables are presented as means±standard deviations or medians (interquartile ranges), as appropriate. Normally distributed continuous variables were compared between the groups using an independent samples *t-*test. Differences in the mean values of continuous variables measured at baseline and after 24 and 72h of the procedure were compared using repeated measures analysis of variance. Non-normally distributed continuous data were compared between the groups using the Mann-Whitney U test. Differences in the mean values of non-normally distributed continuous variables measured at baseline and after 24 and 72 hours of the procedure were compared using the Friedman test. Categorical variables are presented as frequencies (percentages). Comparisons between the groups were performed using the *χ*^2^ test. The Wilcoxon rank-sum test was used for the comparison of rank variable data. Univariate logistic regression analysis was performed in all patients to identify the risk factors for CIN. Sex, age, medical history, heart rate, NYHA cardiac functional classification, levels of biomarkers (such as glutamic oxaloacetate transaminase, glutamic pyruvate transaminase, N-terminal pro-B-type natriuretic peptide, serum albumin, total cholesterol, triglyceride, low-density lipoprotein cholesterol, high-density lipoprotein cholesterol, hemoglobin, glycosylated hemoglobin, UA, SCr, and Cys C), eGFR, volume of CM, and Mehran CIN-risk scores were used for univariate logistic regression analysis. Thereafter, the statistically significant variables in the univariate analysis were used in multivariate analysis to determine the independent risk factors and to calculate the odds ratio (OR). For all tests, a *p* value of <0.05 was considered statistically significant.

## RESULTS

### Baseline characteristics

**Figure 1:**
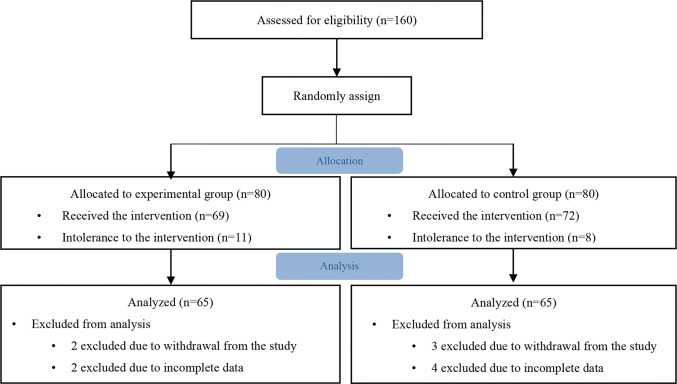
Flow chart of patient selection.

Overall, 160 patients were allocated 1:1 to the experimental (n=80) or control group (n=80). Fifteen patients from both the groups were not included in statistical analysis because of intolerance to intervention, withdrawal from the study, or incomplete data (Figure 1). The baseline characteristics of all patients are shown in Table 1. No significant differences were found in the baseline characteristics, except the triglyceride level and previous history of cerebral infarction, between the two groups (*p*>0.05). The coronary angiography results are summarized in Table 2. No significant difference was found between the two groups in terms of coronary artery lesions, proportion of coronary intervention, and volume of CM (*p*>0.05).

### Patients in the experimental group had significantly lower SCr level and higher eGFR after the procedure than patients in the control group

**Figure 2:**
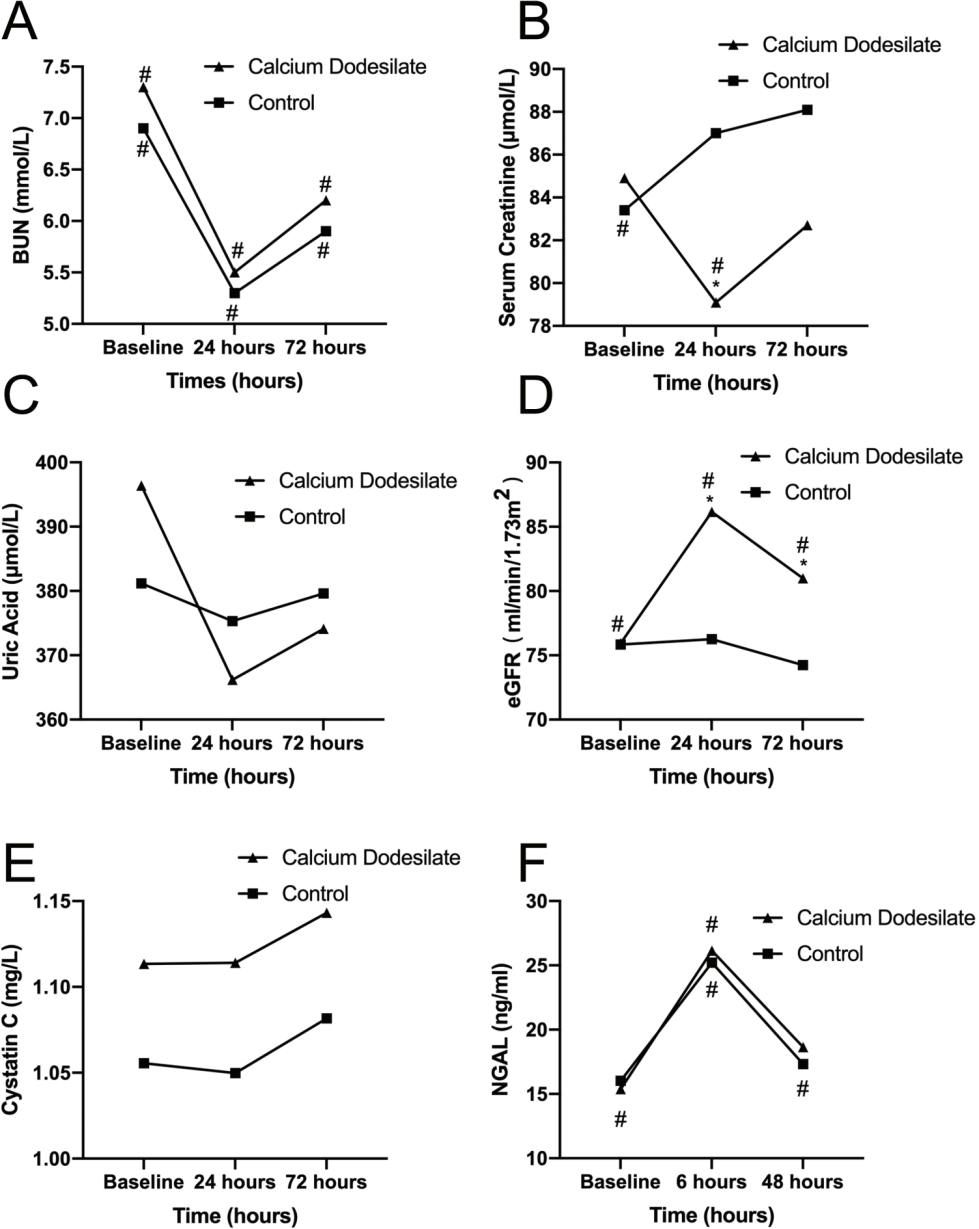
The Comparison of the blood urea nitrogen (A), serum creatinine (B), uric acid (C), eGFR (D), Cystatin C (E), and NGAL (F) levels between the experimental (calcium dobesilate receiving) and control groups. Abbreviations: BUN, blood urea nitrogen; eGFR, estimated glomerular filtration rate; NGAL, neutrophil gelatinase-associated lipocalin *p<0.05, compared with the control group, ^#^ *p*<0.05, compared with the other two measurement time points in the same group.

The levels of BUN, SCr, UA, and eGFR before and 24 and 72h after CAG or PCI are presented in Table 3, and the changes of them are shown in Figure 2A-D. The initial BUN, SCr, and UA level and eGFR were not significantly different between the two groups. At 24 and 72h after CAG or PCI, no significant differences were found in the BUN and UA levels between the two groups (all *p*>0.05). However, the mean SCr level at 24h after the procedure was significantly lower in the experimental group than in the control group (*p*=0.023). Likewise, the eGFR at 24 and 72h after CAG or PCI was significantly higher in the experimental group than in the control group (*p*=0.016). Compared with the levels of the aforementioned biomarkers before and after the procedure, the BUN level decreased in the first 24h after the procedure and thereafter, significantly increased at 72h in both the groups (both *p*<0.001). The SCr level at 24h was significantly lower than those at baseline and 72h after the procedure in the experimental group (*p*=0.004). Meanwhile, the mean SCr levels at 24 and 72h after the procedure were significantly higher than that at baseline in the control group (*p*=0.018). The eGFR at all three measurement time points did not statistically differ in the control group (*p*=0.329). However, the eGFR at 24h after the procedure was significantly higher than that at baseline and 72h after the procedure in the experimental group (*p*<0.001).

### Cys C and NGAL levels were not significantly different between two groups

Table 3 shows the levels of Cys C and NGAL before and after the procedure in the two groups, and Figure 2E-F show the changes of Cys C and NGAL. The Cys C and NGAL levels were not significantly different between the two groups at any of the three measurement time points (all *p*>0.05). In both the groups, a slight increase in the Cys C level was observed at 24 and 72h compared with baseline, without significance. The NGAL level significantly increased at 6h after the procedure (both *p*<0.001) and thereafter, decreased in 48 h in both the groups.

### Difference in changes in all renal function biomarkers, except SCr and eGFR between the two groups were not significant

The changes in the BUN, SCr, UA, Cys C, and NGAL levels and eGFR were calculated by subtracting the baseline values from the peak values measured after the procedure (Table 3). The changes in the SCr level were significantly higher in the control group than in the experimental group (*p*=0.006). However, the changes in the eGFR were significantly lower in the control group than in the experimental group (*p*=0.004). No significant differences were found in the changes in the BUN, UA, Cys C, and NGAL levels between the two groups (all *p*>0.05).

### Incidence of CIN defined by the SCr level, but not by the Cys C level significantly differed between the two groups

A total of 3 (4.6%) patients in the experimental group and 13 (20.0%) in the control group were diagnosed with CIN, when CIN was defined by the SCr level (an increase in the SCr level by 25% or an SCr level of >44 μmol/L). A statistically significant difference was found between the two groups for the incidence of CIN (*p*=0.017) (Table 3). However, when CIN was defined by the Cys C level (an increase in the Cys C level by >25% after the procedure), seven (10.8%) patients were diagnosed with CIN in the two groups. No statistical difference was found between the two groups for the incidence of CIN (*p*=1.000) (Table 3). None of the patients in either group required dialysis therapy.

### Logistic regression analysis showed that calcium dobesilate affected the incidence of CIN defined by the SCr level, but not by the Cys C

Univariate logistic regression analysis was performed in all patients to identify the risk factors for CIN. The results of univariate logistic regression analysis are shown in Table 4. Diuretics, calcium dobesilate, serum albumin level, heart rate, NYHA class III (compared with NYHA class I), and Mehran CIN risk score were significantly associated with the incidence of CIN defined by the SCr level. However, only age, diuretics, NYHA class III (compared with NYHA class I), and Mehran CIN risk score were associated with the incidence of CIN defined by the Cys C level.

Significant variables in univariate analysis were used in multivariate analysis, and the OR was adjusted according to age, sex, volume of CM, baseline SCr level, and baseline eGFR. The risk factor for CIN defined by the SCr level was diuretics. Calcium dobesilate (OR=0.071, 95% CI: 0.012-0.404, *p*=0.003), serum albumin level, and heart rate were protective factors for CIN. The Mehran CIN risk score was the only risk factor for CIN defined by the Cys C level (Table 5).

## DISCUSSION

In this study, calcium dobesilate reduced the incidence of SCr and CIN, as defined by the SCr level after CAG/PCI. However, no significant differences were observed in the levels of Cys C and NGAL, which are the more sensitive biomarkers, between the two groups. In addition, no statistical difference was found between the two groups in CIN defined by the Cys C level. Therefore, the results of this study did not support the effect of calcium dobesilate in preventing CIN in patients with diabetes and CKD.

Previous studies have reported that N-acetylcysteine, statins, and other drugs may prevent CIN (9-11). However, to the best of our knowledge, no study has reported the preventive effect of calcium dobesilate on CIN. This study was the first randomized controlled trial to assess the effect of calcium dobesilate in preventing CIN in patients with diabetes and CKD.

The results showed a significant difference in the SCr level and eGFR after the application of calcium dobesilate compared with the control group. In addition, the incidence of CIN defined by the SCr level was significantly lower in patients pretreated with calcium dobesilate. The use of calcium dobesilate was also an independent protective factor for CIN. Thus, the above results indicate the preventive effect of calcium dobesilate on CIN.

To confirm the preventive effect of CIN, we measured the Cys C and NGAL levels to evaluate kidney function impairment. Cys C, a 13kDa non-glycosylated protein, is a sensitive biomarker of kidney function (27). The serum Cys C level has been shown to be a better endogenous marker of GFR and more sensitive for rapid detection of acute changes in renal function, especially after PCI, than the SCr level (28). In patients with CIN, the level of Cys C increases within 24h after CAG, which is earlier than the time required for the SCr level to increase (29). NGAL is a 25 kDa glycoprotein of the lipocalin superfamily. Recently, NGAL has been considered a predictor of acute kidney injury because the serum and urine levels of NGAL increase before the SCr level increases (30). Studies have revealed that the NGAL level increases at 2h after the application of CM, whereas the SCr level increases at least after 12-24h of the application of CM (29). According to a meta-analysis by Briasoulis et al., the NGAL level in the serum and urine have high specificity and sensitivity in the early diagnosis of CIN (31). However, no significant difference was found between the experimental and control groups for the Cys C and NGAL levels after the procedure. Meanwhile, the incidence of CIN defined by the Cys C level was similar in both the groups. Risk factor analysis for CIN revealed that the Mehran score alone was an independent risk factor for CIN. Therefore, these results suggest that calcium dobesilate barely prevents CIN.

Considering the aforementioned factors, it may be reductive to the reducibility of calcium dobesilate, which consumes hydrogen peroxide (H_2_O_2_) in the Trinder reaction of the sarcosine oxidase assay, inhibiting the oxidative coloration of chromogenic phenol and thus, interfering with the detection of SCr, causing a negative interference (32). Compared with the control group, SCr significantly decreased after the application of calcium dobesilate. The eGFR calculated based on the SCr level and CIN (as defined by the SCr level) were interfered, which led to the illusion of CIN prevention by calcium dobesilate. However, the results of other renal function biomarkers suggested that calcium dobesilate may not have the capability of protecting renal function and preventing CIN.

However, although many studies have confirmed the renal protective effect of calcium dobesilate, it cannot reduce the occurrence of CIN through short-term application. Calcium dobesilate is used for the treatment of diabetic retinopathy and diabetic nephropathy (33,34). Studies have suggested that the therapeutic effect of calcium dobesilate depends on the reduction of endothelin levels (33) and thereby, dilation of blood vessels; antioxidant effects (35); and amelioration of microinflammation (36,37). In addition, studies have demonstrated that calcium dobesilate prevents acute renal injury induced by streptomycin and gentamicin in animals (35). Therefore, the pharmacological effect of calcium dobesilate in the pathogenesis of CIN might be targeted prevention of CIN. Although the pathogenesis of CIN has not yet been clarified, it is believed that the pathological mechanism of CIN might be related to the hemodynamic changes caused by contrast agent-induced vasoconstriction, which further leads to renal ischemia and a decrease in the eGFR (15). In addition, many studies have shown that CM is cytotoxic and augments reactive oxygen species and renal oxidative stress, which can cause autophagy or apoptosis of renal tubular epithelial cells (16,38), resulting in renal damage. Furthermore, studies have shown that CIN is associated with a systemic inflammatory response and CRP and other inflammatory biomarkers are significantly associated with the incidence of CIN (17,18). According to a study by Zhou et al., calcium dobesilate might have angio-protective properties and protect endothelial cells partly by ameliorating high glucose-induced endothelial dysfunction and inflammation (37); therefore, calcium dobesilate might be used to prevent CIN through this mechanism.

Hence, we speculated that calcium dobesilate should have a preventive effect against CIN. However, this study failed to show the preventive effect, which may be because of the following reasons: 1. the renal protective effect of calcium dobesilate is weaker than that of hydration therapy, and the effect of calcium dobesilate is covered up. Therefore, the effect of calcium dobesilate can be further verified in individuals who cannot tolerate hydration therapy for cardiac insufficiency. 2. Because of the limited sample size, this study did not explore the preventive effect in different pretreatment periods and dosages of calcium dobesilate. Hence, further research with a longer medication duration or increased dosage might lead to better results. 3. The results without significance may be related to the limited sample size of this study, and the conclusions of this study can be further confirmed by increasing the sample size.

This study has some limitations. First, sample size of the study was relatively small. Second, our study was not a multicenter, double-blind placebo-control study. In the future, a multicenter, double-blind placebo-control study with a large sample size should be conducted to confirm this conclusion.

## CONCLUSION

This study found that calcium dobesilate does not prevent CIN in patients with diabetes and chronic renal failure.

## AUTHOR CONTRIBUTIONS

Zhang H was responsible for the data collection, statistical analysis and manuscript preparation. Guo SH was responsible for the data interpretation, manuscript preparation and literature search. Xue ZK, Zhang YR, Wang JR and Ashraful-Haque SZ were responsible for the data collection and literature search. Che JJ and Liu T were responsible for the study design and statistical analysis. Tao HY was responsible for the statistical analysis. Li GP and Rha SW were responsible for the study design. Chen KY was responsible for the study design, data interpretation and funds collection.

## Figures and Tables

**Table 1:** Baseline clinical characteristics of the patients.

Variables	Experimental group (n=65)	Control group (n=65)	*p* value
Age (years)	68±9	67±9	0.558
Male sex, *n* (%)	40 (61.5)	34 (52.3)	0.376
Height (cm)	167.4±6.7	165.1±8.0	0.075
Weight (kg)	71.3±11.0	68.8±12.6	0.224
Heart rate (bpm)	70±9	72±10	0.463
Cardiac functional classification			0.160
NYHA I, *n* (%)	55 (84.6)	48 (73.8)	
NYHA II, *n* (%)	9 (13.8)	15 (23.1)	
NYHA III, *n* (%)	1 (1.5)	2 (3.1)	
Comorbidities			
Hypertension, *n* (%)	51 (78.5)	53 (81.5)	0.827
Previous MI, *n* (%)	6 (9.2)	14 (21.5)	0.087
Atrial fibrillation, *n* (%)	2 (3.1)	2 (3.1)	1.000
Peptic ulcer, *n* (%)	7 (10.8)	6 (9.2)	1.000
COPD, *n* (%)	5 (7.7)	5 (7.7)	1.000
Previous cerebral infarction, *n* (%)	4 (6.2)	13 (20.0)	0.035
ALT (U/L)	17.8 (14.3, 28.9)	18.5 (12.6, 23.7)	0.382
AST (U/L)	17.1 (14.3, 21.6)	16.2 (12.9, 18.9)	0.097
Total cholesterol (mmol/L)	4.58±1.07	4.51±1.02	0.729
Triglyceride (mmol/L)	1.97 (1.39, 3.26)	1.40 (1.16, 2.33)	0.001
HDL-C (mmol/L)	1.03±0.25	1.09±0.28	0.165
LDL-C (mmol/L)	2.80±0.88	2.74±0.83	0.716
Serum albumin (g/L)	42.9±3.8	42.2±4.0	0.255
NT-proBNP (ng/mL)	58.8 (22.8, 189.5)	64.5 (16.2, 194.5)	0.995
FBG (mmol/L)	7.47 (6.01, 9.36)	6.70 (5.67, 8.09)	0.058
HbA1c (%)	7.1 (6.5, 8.5)	6.7 (6.4, 7.7)	0.084
CK (U/L)	67.2 (51.4, 124.9)	61.2 (45.5, 115.6)	0.615
CK-MB (U/L)	10.4 (8.2, 14.4)	10.0 (6.0, 13.8)	0.430
Cardiac troponin I (ng/L)	0.003 (0.001, 0.010)	0.004 (0.001, 0.026)	0.637
Hemoglobin (g/L)	138.0±17.6	136.9±17.3	0.729
LVEF (%)	57.5±9.1	58.9±9.0	0.395
Drug history			
Statins, *n* (%)	63 (96.9)	60 (92.3)	0.440
ACEIs/ARBs, *n* (%)	47 (72.3)	47 (72.3)	1.000
β-blockers, *n* (%)	50 (76.9)	43 (66.2)	0.243
Diuretics, *n* (%)	16 (24.6)	9 (13.8)	0.181
CCBs, *n* (%)	25 (38.5)	34 (52.3)	0.158
Mehran CIN risk score, *n* (%)	5 (4,8)	6 (5,10)	0.367

Data are expressed as *x̄*±*s, M (P25, P75)*, and frequencies (%). Abbreviations: ACEIs, angiotensin-converting enzyme inhibitors; ALT, glutamic oxaloacetate transaminase; ARB, angiotensin II receptor blocker; AST, glutamic pyruvate transaminase; CCBs, calcium channel blockers; CIN, contrast-induced nephropathy; CK, creatine kinase; CK-MB, creatine kinase isoenzyme MB; COPD, chronic obstructive pulmonary disease; FBG, fasting plasma glucose;, HbA1c glycosylated hemoglobin; HDL-C, high-density lipoprotein-cholesterol; LDL-C, low-density lipoprotein-cholesterol; LVEF, left ventricular ejection fraction; MI, myocardial infarction; NT-proBNP, N-terminal pro-B-type natriuretic peptide; NYHA, New York Heart Association functional classification.

**Table 2:** Comparison of coronary artery angiography results between the two groups.

Variables	Experimental group (n=65)	Control group (n=65)	*p* value
Lesions of coronary artery			0.376
Normal coronary artery, *n* (%)	5 (7.7)	5 (7.7)	
Single vessel disease, *n* (%)	9 (13.8)	11 (16.9)	
Double vessel disease, *n* (%)	15 (23.1)	19 (29.2)	
Triple vessel disease, *n* (%)	36 (55.4)	30 (46.2)	
Proportion PCI, *n* (%)	36 (55.4)	30 (46.2)	0.380
Volume of contrast media (mL)	110 (60, 150)	70 (55, 140)	0.507

Data are presented as *M (P25, P75)* and frequencies (%). Abbreviations: PCI, percutaneous coronary intervention.

**Table 3:** Comparison of renal function biomarkers between two groups.

Variables	Experimental group (n=65)	Control group (n=65)	*p* value
BUN (mmol/L)			
Baseline	7.3±2.2*	6.9±2.2*	0.320
24h after procedure	5.5±2.0*	5.3±1.7*	0.531
72h after procedure	6.2±2.2*	5.9±2.1*	0.565
*p* among levels of three times measurement	<0.001	<0.001	
ΔBUN (mmol/L)	−0.8±1.9	−0.7±1.8	0.753
SCr (μmol/L)			
Baseline	84.9±21.1	83.4±19.4*	0.673
24h after procedure	79.1±19.6*	87.0±19.3	0.023
72h after procedure	82.7±21.0	88.1±20.2	0.136
*p* among levels of three times measurement	0.004	0.018	
ΔSCr (μmol/L)	0.4±14.6	7.7±14.9	0.006
UA (μmol/L)			
Baseline	396.4±106.1**	381.2±115.1	0.436
24h after procedure	366.2±110.1**	375.3±107.4	0.635
72h after procedure	374.1±115.0	379.6±97.3	0.768
*p* among levels of three times measurement	0.016	0.737	
ΔUA (μmol/L)	0.0±79.4	13.2±75.6	0.336
eGFR (mL/min/1.73m^2^)			
Baseline	76.65±15.56*	75.85±14.23	0.760
24h after procedure	86.88±19.93*	76.26±15.73	0.001
72h after procedure	81.85±19.15*	74.25±16.22	0.016
*p* among levels of three times measurement	<0.001	0.329	
ΔeGFR (mL/min/1.73m^2^)	2.57±13.33	−4.01±12.23	0.004
Cystatin C (mg/L)			
Baseline	1.00 (0.84, 1.25)	0.98 (0.83, 1.24)	0.575
24h after procedure	0.99 (0.85, 1.29)	1.00 (0.82, 1.15)	0.703
72h after procedure	1.01 (0.87, 1.33)	1.04 (0.86, 1.24)	0.679
*p* among levels of three times measurement	0.362	0.065	
ΔCys C (mg/L)	0.04 (−0.01, 0.12)	0.02 (−0.04, 0.13)	0.949
NGAL (ng/mL)			
Baseline	13.00 (11.10, 20.00)	15.70 (12.04, 19.21)	0.407
6h after procedure	23.03 (15.63, 32.91)	20.81 (15.54, 30.49)	0.798
48h after procedure	18.12 (12.41, 21.31)	15.65 (13.3, 18.93)	0.250
*p* among levels of three times measurement	<0.001	<0.001	
ΔNGAL (ng/mL)	6.58 (1.30, 16.46)	5.1 (2.01, 14.44)	0.694
CIN defined by SCr, *n* (%)	3 (4.6)	13 (20.0)	0.014
CIN defined by Cys C, *n* (%)	7 (10.8)	7 (10.8)	1.000

Data are presented as *x̄*±*s* and frequencies (%). Δ: for all biomarkers, the peak value measured after the procedure minus the baseline value. Abbreviations: BUN, blood urea nitrogen; CIN, contrast-induced nephropathy; eGFR, estimated glomerular filtration rate; NGAL, neutrophil gelatinase-associated lipocalin; SCr, serum creatinine; UA, uric acid. *p<0.05 compared with the other two groups; **p<0.05 compared with each other.

**Table 4:** Univariate logistic regression analysis for the incidence of CIN.

	CIN defined by serum creatinine level			CIN defined by cystatin C level		
	*p* value	OR	95% CI	*p* value	OR	95% CI
Sex	0.954	0.969	0.337-2.784	0.557	1.412	0.446-4.474
Age	0.162	1.046	0.982-1.113	0.048	1.074	1.001-1.153
Height	0.442	0.973	0.906-1.044	0.385	1.034	0.959-1.116
Weight	0.215	0.970	0.925-1.018	0.502	1.016	0.970-1.064
Cardiac functional classification						
NYHA I	0.084	Reference	-	0.063	Reference	-
NYHA II	0.798	1.195	0.306-4.663	0.836	0.845	0.173-4.136
NYHA III	0.026	16.727	1.400-199.856	0.021	18.600	1.546-223.779
Previous hypertension	0.174	4.213	0.530-33.468	0.888	0.907	0.234-3.518
Previous MI	0.691	1.317	0.339-5.115	0.159	2.500	0.699-8.938
Previous COPD	0.448	1.893	0.365-9.823	0.999	0.000	-
Statins	0.999	0.000	-	0.759	0.709	0.079-6.359
ACEI/ARB	0.797	1.171	0.352-3.899	0.581	1.458	0.382-5.561
β−blockers	0.792	0.859	0.276-2.667	0.539	1.520	0.399-5.793
Diuretics	0.044	3.200	1.033-9.910	0.024	3.829	1.192-12.302
Calcium dobesilate	0.014	0.194	0.052-0.716	1.000	1.000	0.330-3.032
CCB	0.888	0.927	0.323-2.661	0.444	0.638	0.201-2.019
AST	0.684	0.992	0.957-1.029	0.929	1.002	0.952-1.056
ALT	0.801	0.993	0.937-1.052	0.956	1.001	0.971-1.031
Serum albumin	0.018	0.838	0.725-0.970	0.087	0.878	0.757-1.019
NT-proBNP	0.933	1.000	0.999-1.001	0.640	1.000	1.000-1.001
HbA1c	0.285	0.783	0.500-1.226	0.497	1.119	0.808-1.550
Cardiac troponin I	0.594	0.738	0.241-2.258	0.613	0.680	0.152-3.038
Hemoglobin	0.166	0.979	0.950-1.009	0.614	0.992	0.961-1.024
Baseline BUN	0.931	0.990	0.780-1.255	0.997	1.000	0.780-1.284
Baseline SCr	0.582	0.992	0.965-1.020	0.920	0.999	0.971-1.027
Baseline UA	0.147	0.996	0.991-1.001	0.092	0.995	0.989-1.001
Baseline CysC	0.253	2.063	0.596-7.141	0.654	0.693	0.139-3.452
Baseline eGFR	0.970	1.001	0.966-1.037	0.941	0.999	0.962-1.036
Baseline NGAL	0.629	1.024	0.929-1.130	0.600	0.971	0.870-1.084
LVEF	0.377	0.976	0.924-1.030	0.460	0.978	0.924-1.037
Artery fibrillation	0.294	3.733	0.319-43.707	0.240	4.385	0.372-51.737
Heart rate	0.022	0.917	0.852-0.988	0.211	0.956	0.891-1.026
Lesion of coronary artery						
Triple vessel disease	0.615	Reference	-	0.818	Reference	-
Double vessel disease	0.453	1.554	0.492-4.909	0.399	1.724	0.486-6.120
Single vessel disease	0.378	0.382	0.045-3.251	0.454	1.765	0.399-7.806
Normal coronary artery	0.847	0.806	0.090-7.228	0.999	0.000	-
PCI	0.262	1.852	0.631-5.436	0.951	1.035	0.341-3.138
Volume of CM	0.999	1.000	0.990-1.010	0.499	1.004	0.993-1.014
Mehran CIN risk score	0.027	1.157	1.017-1.316	0.020	1.173	1.026-1.342

Abbreviations: ACEI, angiotensin-converting enzyme inhibitor; ALT, glutamic oxaloacetate transaminase; ARB, angiotensin II receptor blocker; AST, glutamic pyruvate transaminase; BUN, blood urea nitrogen; CCB, calcium channel blocker; CI, confidence interval; CIN, contrast-induced nephropathy; CM, contrast medium; COPD, chronic obstructive pulmonary disease; Cys C, cystatin C; eGFR, estimated glomerular filtration rate;, HbA1c glycosylated hemoglobin; LVEF, left ventricular ejection fraction; MI, myocardial infarction; NGAL, neutrophil gelatinase-associated lipocalin; NT-proBNP, N-terminal pro-B-type natriuretic peptide; NYHA, New York Heart Association functional classification; OR, odds ratio; PCI, percutaneous coronary intervention; SCr, serum creatinine; UA, uric acid.

**Table 5:** Multivariate logistic regression analysis for occurrence of CIN.

	*p* value	OR	95% CI*
CIN defined by SCr level			
Diuretics	0.015	6.505	1.436-29.473
Calcium dobesilate	0.003	0.071	0.012-0.404
Serum albumin	0.046	0.846	0.718-0.997
Heart Rate	0.005	0.877	0.800-0.961
CIN defined by Cys C level			
Mehran CIN risk score	0.020	1.173	1.026-1.342

Abbreviations: CI, confidence interval; CIN, contrast-induced nephropathy; Cys C, cystatin C; OR, odds ratio; SCr, serum creatinine. *Odds ratios were adjusted according to age, sex, baseline estimated glomerular filtration rate, baseline SCr, baseline Cys C, heart rate, hemoglobin, volume of contrast medium, and Mehran CIN risk score.
